# Hymenoptera venom allergy: How negative testing after five years of immunotherapy informs treatment discontinuation 

**DOI:** 10.5414/ALX02593E

**Published:** 2025-12-19

**Authors:** Matteo Cavara, Alice Botta, Alessandra Chiei Gallo, Eleonora Bono, Christian P. Ratti, Enrico Iemoli, Valeria G.R. Ortolani

**Affiliations:** Allergy and Clinical Immunology Unit, ASST Fatebenefratelli Sacco, Sacco Hospital, Milan, Italy

**Keywords:** hymenoptera venom allergy, venom immunotherapy, anaphylaxis

## Abstract

Background: Hymenoptera venom allergy represents a potentially life‐threatening condition. Venom immunotherapy (VIT) is the treatment of choice in patients with systemic reactions. Case presentation: We report the case of a 65‐year‐old male, who experienced systemic reactions following hymenoptera stings. In vitro and in vivo allergy tests revealed sensitization predominantly to *Polistes dominula* and specific VIT was initiated. During VIT, the patient experienced two subsequent stings with only local reactions. After 5 years of VIT, repeated serological evaluation and skin tests turned negative, leading to the decision to discontinue therapy. Conclusion: This case underlines the efficacy and safety of VIT in patients with severe hymenoptera venom allergy in preventing systemic reactions. A comprehensive diagnostic work-up, including laboratory and skin tests, is crucial in the management and subsequent evaluation of therapeutic efficacy.

## Introduction 

Hymenoptera venom allergy affects up to 7.5% of adults and can cause severe systemic reactions, including anaphylaxis. Accurate diagnosis based on detailed clinical history as well as in vitro and skin testing is essential to identify sensitized patients [1]. Venom immunotherapy (VIT) is the only effective treatment to prevent future systemic reactions, with a success rate exceeding 90% [2]. Although recent studies have confirmed that VIT efficacy persists after discontinuation, supporting the decision to end therapy in selected patients after five years, questions about the optimal treatment duration and discontinuation criteria remain unresolved [3]. This issue is particularly challenging for patients who, thanks to effective environmental prophylaxis, are not re-exposed to stings; without such re-exposure, it is difficult to confirm ongoing protection, complicating the decision to stop therapy. 

## Case presentation 

A 65-year-old male firefighter, presented to our allergy unit in 2019 with a history of three reactions following hymenoptera stings. His medical history included hypertension, treated with telmisartan 20 mg daily, and psoriatic arthritis in remission. He was an ex-smoker. In 1994, he was stung by a honeybee (*Apis mellifera*) on the right forearm resulting in a large local reaction (LLR) managed with oral corticosteroids and oral antihistamines. Following that, in August 2018, a probable European hornet (*Vespa crabro*) sting led to dizziness without any objective findings of systemic reaction. Lastly, in October 2018 a sting by an unidentified vespid caused generalized urticaria and dizziness, which led the patient to call for an ambulance. Paramedics found hypotension (BP 70/50 mmHg). Serum tryptase measured 21.3 µg/mL 1 hour post reaction and 8.1 µg/mL at baseline, after 72 hours, confirming mast cell activation (Δ ≥ 20% of serum basal tryptase + 2 µg/L). Serum tryptase levels were quantitatively measured using an automated fluoroenzyme immunoassay (Thermo Fisher Scientific, Uppsala, Sweden) and 11.4 μg/L was considered the normal upper limit, according to the manufacturer’s instructions. The REMA (Spanish Network on Mastocytosis) score was 1, indicating no need for further investigations to rule out systemic mastocytosis [4]. 

Specific IgE testing showed sensitization to *Vespula spp.* (1.69 kUA/L), *Polistes spp.* (1.96 kUA/L), and *Vespa crabro* (0.23 kUA/L) venoms, but not to *Apis mellifera* venom. Molecular testing was positive for Ves v1 (1.58 kUA/L), Ves v5 (0.24 kUA/L), and Pol d5 (0.48 kUA/L). IgEs for MUXF3 (CCD) were negative. Serum-specific IgE were quantitatively measured using a commercial automated fluoroenzyme immunoassay (Thermo Fisher Scientific), and according to the manufacturer’s instructions, values greater than 0.10 kUA/L were considered positive. 

Skin testing was performed with *Apis mellifera*, *Vespula spp*., *Polistes dominula*, and *Vespa crabro* venom extracts (Anallergo, Florence, Italy). For all venoms, skin prick tests (SPTs) were carried out at a concentration of 100 µg/mL, followed by intradermal tests (IDTs) at concentrations of 0.1 µg/mL and 1 µg/mL. Skin testing revealed a positive IDT to *Polistes dominula* at both 0.1 µg/mL and 1 µg/mL. IDTs were performed by administering 0.02 mL of the allergen extract into the dermis, inducing the formation of a wheal ~ 3 mm in diameter. The reading was carried out after 20 minutes, and a positive result was defined as an increase of at least 3 mm in the mean diameter of the initial wheal, accompanied by surrounding erythema [5]. 

Analysis of the patient’s history indicates that the severe systemic reaction was triggered by a vespid sting, excluding *Apis mellifera* and *Vespa crabro*, previously identified by the patient. Serological tests revealed higher specific IgE levels for *Polistes spp.* and *Vespula spp.*, suggesting a predominant sensitization to these vespids over *Vespa crabro* and *Apis mellifera*. The weakly positive result for *Vespa crabro* may reflect residual sensitization from a previous sting. Intradermal skin tests were positive for *Polistes dominula* indicating sensitization to this species. Based on these findings, VIT with an L-tyrosine-adsorbed subcutaneous immunotherapy for *Polistes dominula* venom (Anallergo) was initiated in June 2019 with a clustered protocol. The protocol was followed according to the manufacturer’s plan (Table 1), with the maintenance dose of 1 mL of 100 µg/mL hymenoptera venom reached within 8 weeks, without pretreatment with antihistamines. On each day of up-dosing, the injections were administered at 30-minute intervals followed by a 3-hour observation period after the final injection. The first maintenance injection was given 4 weeks later. Each maintenance dose was followed by a 30-minute observation period. During the second year, the dosing interval was extended from every 4 to every 6 weeks, and from the third year onward, it was further extended to every 8 weeks. The safety of the clustered protocol has been well documented in the medical literature [6]. 

During both the dose-escalation and maintenance phases, the patient did not experience local or systemic reactions. He was stung twice during the course of therapy, first by a vespid after 1 year, and subsequently by a European hornet (*Vespa crabro*) after 2.5 years, experiencing only mild local reactions. After 5 years, serum-specific IgE levels (commercial automated fluoroenzyme immunoassay, Thermo Fisher Scientific) were undetectable (< 0.10 kUA/L), and skin tests (SPTs and IDTs) for *Apis mellifera*, *Vespula spp.*, *Polistes dominula*, and *Vespa crabro* venom extracts (Anallergo) were negative, prompting VIT discontinuation, in agreement with the patient. It is most likely that all IgE and skin tests turned negative because *Polistes* venom was the primary sensitizing venom. VIT with *Polistes dominula*-induced specific immune tolerance, and the cross-reactive sensitizations to other vespids gradually waned, leading to the complete loss of both serological and skin test reactivity. 

## Discussion 

This case underscores several key aspects relevant to the management and discontinuation of VIT. 

Current literature supports a VIT course of 3 to 5 years as effective in both adults and children. Notably, a 1-year regimen is insufficient for ~ 25% of treated patients [7]. Data indicate that after 3 years of VIT, between 83% and 100% of patients remain protected against subsequent stings during the following 1 – 3 years, and evidence suggests that 80 – 90% of patients will not experience a systemic reaction if VIT is stopped after this period. Moreover, treatment durations of 5 years or more have been associated with prolonged post-therapy protection [5]. The minimum recommended duration is 3 years, with 5 years being generally advised; lifelong treatment should be considered in the presence of specific risk factors [8]. 

Even though complete negativization of skin tests and specific serum IgE levels may offer reassurance, these results are uncommon [9]. In many cases, even in the absence of re-stings, often due to strict environmental avoidance, specific IgE levels remain elevated at the end of the 5-year treatment period, typically reduced by 58 – 70% compared to baseline [5]. An increasing body of evidence indicates that despite a persisting positive skin test, many patients remain clinically protected [9]. However, there are no specific tests to predict which patients might relapse after stopping VIT. Relapse is generally less likely with a 5-year treatment duration than with only 3 years and is less common in younger children compared to adolescents or adults [10]. 

For patients with high-risk features, continuation of VIT beyond 5 years should be individualized according to the patient’s clinical characteristics. Extended or even indefinite VIT is recommended in patients with recurrent systemic allergic reactions during maintenance therapy or if a systemic reaction occurs after a sting by the culprit insect while on maintenance therapy. In certain situations, prolongation of VIT may also be justified for quality-of-life reasons, especially when patients are exposed to a high risk of stings, such as beekeepers. Long-term therapy may further be indicated in patients with mastocytosis or persistently elevated baseline tryptase levels (> 20 µg/L), in those who have experienced cardiovascular or respiratory arrest due to hymenoptera sting anaphylaxis, or in the presence of other exceptionally strong risk factors, such as hereditary α-tryptasemia [11]. 

Criteria suggested for stopping VIT include a finite treatment duration, commonly 3 – 5 years, a decrease in serum venom-specific IgE antibodies to undetectable levels, or conversion to a negative skin test response. Although some authors advocate for repeat testing every 3 – 5 years, negative results are uncommon until after 5 years, and repeat skin or serum IgE testing is not mandatory when considering discontinuation. In a few cases, patients with prior severe anaphylaxis, even after more than 5 years of immunotherapy and negative in vitro or skin test responses, have later experienced systemic reactions upon re-sting [12]. These observations indicate that decisions regarding discontinuation should not rely solely on serological markers or skin test reactivity. 

The live sting challenge is the only method that can definitively demonstrate clinical tolerance and guide decisions regarding the safe discontinuation of VIT. While highly informative, it is not routinely performed in many centers due to logistical and safety considerations, as it involves deliberate exposure to venom with the potential to trigger systemic allergic reactions. Performing a sting challenge safely requires appropriate emergency infrastructure, consequently, many allergy units rely on alternative assessments such as detailed clinical history, in vivo skin testing, and in vitro measurement of specific IgE levels to evaluate the efficacy of VIT. Importantly, only a non-tolerated sting challenge can definitively indicate failure of VIT, which may necessitate adjustment of the maintenance dose or prolongation of therapy [13]. 

A thorough evaluation of risk factors is crucial when deciding whether to extend or discontinue VIT. For instance, in patients with mast cell disorders, many experts advocate for lifelong VIT, even in the absence of definitive scientific evidence, due to an increased risk of systemic reactions. For patients without such high-risk features, the decision to extend VIT beyond 5 years should be made collaboratively, considering factors such as the severity of previous systemic reactions and the patient’s quality of life. In all cases, long-term follow-up is essential. Patients not undergoing VIT but prescribed an epinephrine auto-injector should receive regular follow-up, especially following re-stings, and periodic re-evaluation (e.g., every 2 years) with allergy testing. Similarly, patients on VIT should be assessed at 3- and 5-year intervals through both skin testing and measurement of specific IgE levels, particularly if any systemic reactions occur during therapy. Post-discontinuation follow-up should include re-assessment at each prescription renewal of auto-injectable epinephrine, accompanied by refresher training on its use [5]. 

## Conclusion 

This case underscores several key aspects of VIT management and discontinuation. First, individualized risk stratification, based on a detailed clinical history, supported by serological and skin test evaluations, is essential to appropriately initiate and monitor VIT. The long-term efficacy of VIT is reflected in the absence of systemic reactions upon re-sting during therapy, in line with published data. However, the decision to discontinue VIT is particularly challenging in patients who are not re-stung due to effective environmental prophylaxis. Although complete negativization of allergologic tests would ideally support safe discontinuation, this outcome is infrequent. Consequently, decisions regarding the cessation of VIT must be tailored to the individual, integrating clinical findings, risk factor analysis, and patient preferences, in accordance with EAACI recommendations and current literature. In summary, a multimodal diagnostic approach, including patient medical history, serological assays, and skin testing, is vital for the initiation and management of VIT. While VIT has proven highly effective in preventing systemic reactions, its discontinuation should be approached with caution. Long-term, individualized follow-up remains the cornerstone of optimal management in patients with hymenoptera venom allergy. 

## Acknowledgment 

We would like to acknowledge our patient for his willingness to publish his case. 

## Consent for publication 

Written consent for publication was obtained from the patient. 

## Authors’ contributions 

MC and AB have contributed equally to the conception, realization and writing the paper. CPR, EB and ACG have contributed to literature data collection. VGRO and EI have revised the manuscript. 

## Conflict of interest 

The authors declare that they have no conflict of interest. 

## Funding 

The authors declare that no funding was received for the present study. 

**Table 1.** Cluster protocol.

**Day**	**Extract concentration**	**Dose per injection**
Day 1	1 µg/mL	0.1 mL
		0.3 mL
		0.6 mL
		1.0 mL
Day 7	10 µg/mL	0.2 mL
		0.4 mL
Day 14	10 µg/mL	0.6 mL
		1.0 mL
Day 21	100 µg/mL	0.1 mL
		0.2 mL
Day 28	100 µg/mL	0.3 mL
		0.3 mL
Day 35	100 µg/mL	0.5 mL
		0.5 mL
Day 49	100 µg/mL	1.0 mL
